# Decorating gold nanostars with multiwalled carbon nanotubes for photothermal therapy

**DOI:** 10.1098/rsos.180159

**Published:** 2018-08-15

**Authors:** Yuting Zhu, Quanmei Sun, Yingzhu Liu, Tao Ma, Lei Su, Sidi Liu, Xiaoli Shi, Dong Han, Feng Liang

**Affiliations:** 1 The State Key Laboratory for Refractories and Metallurgy, Institute of Advanced Materials and Nanotechnology, School of Chemistry and Chemical Engineering, Wuhan University of Science and Technology, Wuhan 430081, People's Republic of China; 2 CAS Center for Excellence in Nanoscience, National Center for Nanoscience and Technology, Beijing 100190, People's Republic of China; 3 School of Future Technology, University of Chinese Academy of Sciences, Beijing 100049, People's Republic of China; 4 School of Chemistry and Biological Engineering, University of Science and Technology, Beijing 100083, People's Republic of China

**Keywords:** gold nanostars, multiwalled carbon nanotubes, photothermal therapy, cancer cell

## Abstract

Gold nanoparticles and carbon nanotubes have attracted substantial attention in recent years for their potential applications in photothermal therapy (PTT) as an emerging breakthrough in cancer treatment. Herein, a hybrid nanomaterial of gold nanostars/multiwalled carbon nanotubes (MWCNTs) was synthesized by two-step reduction via the control of several synthetic conditions such as the reducing agent, pH value, concentration and ratio of reagents. The material shows good biocompatibility and high photothermal conversion efficiency, demonstrating its applicability in PTT. The lack of surfactant in the synthesis process made the hybrid nanomaterial cell-friendly, with no effects on viability *in vitro*. The MWCNT/gold nanostars hybrid nanomaterial presented 12.4% higher photothermal efficiency than gold nanostars alone and showed a 2.4-fold increase over gold nanospheres based on a heating test under 808 nm laser irradiation. Moreover, the MWCNTs/gold nanostars at low concentration (0.32 nM) exhibited remarkably improved photothermal cancer cell-killing efficacy, which may be attributed to the surface plasmon resonance absorption of the gold nanostars and the combined effects of enhanced coupling between the MWCNTs and gold nanostars. Collectively, these results demonstrate that the MWCNTs/gold nanostars developed herein show prominent photothermal value, and thus may serve as a novel photothermal agent for cancer therapy.

## 1. Introduction

Cancer is currently one of the leading causes of death worldwide, despite significant advances in treatment modalities and progress in understanding cancer biology [1]. Photothermal therapy (PTT) is a novel and important potential method in cancer treatment, which has been widely studied and developed in recent years owing to its significant advantages over conventional cancer therapies, including its non-invasive nature and minimal side effects [2–6]. In particular, because the tissue penetration depth of near-infrared (NIR) light is limited to 1–2 cm, PTT is considered to be more suitable for cancers of relatively shallow tissues such as melanoma [7,8]. PTT is based on photothermal conversion agents that can ablate cancer cells or tissues through converting light into thermal energy [9]. A variety of PTT agents have been reported to date, such as magnetic nanoparticles [10], carbon nanomaterials [11] and metal nanoparticles [12–15]. These nanoparticles can easily accumulate in tumours passively through the enhanced permeability and retention (EPR) effect, or actively through guidance of surface-conjugated targeting molecules by intravenous injection [16]. Among them, gold nanoparticles represent an ideal PTT agent, and are the most widely used owing to their facile preparation, biocompatibility, chemical inertness and unique optical property of localized surface plasmon resonance (LSPR) with high photothermal conversion efficiency; moreover, the LSPR peak could be easily tuned from the visible to the NIR region simply by altering the shape and size of the particles [17]. As the therapeutic window for tissues ranges from 650 to 950 nm, gold nanospheres are not considered to be promising PTT agents *in vivo* given their peak absorption limitation of 400–600 nm [18,19]. In recent years, a variety of gold nanoparticles with controlled size and morphology have been widely developed for biological applications, which exhibit well-defined LSPR peak absorptions, such as gold nanoshells [20], gold nanorods [21], gold nanostars [22–26] and gold nanocages [27].

Among the various types of non-spherical gold nanoparticles, gold nanostars, with a multiple ‘tips’ structure, could offer unique plasmon properties with higher NIR absorption than other gold nanoparticles [23]. Accordingly, gold nanostars have been widely used in various biomedical applications, including surface-enhanced Raman spectroscopy, photodynamic therapy [28–30], photoacoustic imaging [31], biosensor fabrication [32–34] and PTT [35–38]. The plasmon band shift is determined by the branches, branch number, branch length and overall size of the nanostars. However, the traditional synthesis method of gold nanostars often requires use of a surfactant such as cetyltrimethylammonium bromide (CTAB) or polyvinylpyrrolidone with potential for causing damage to the body [39]. Therefore, the development of a method with a simple process and few chemical reagents to synthesize abundant long-tip nanostars is extremely important to improve and expand the use of PTT in cancer therapy.

One potential approach for the preparation of PTT agents is the use of multiwalled carbon nanotubes (MWCNTs), which show impressive ability to convert NIR radiation into heat and have good biocompatibility that can be exploited for the fabrication of hybrid nanomaterials [40,41]. Indeed, *in vitro* studies demonstrated that incorporation of MWCNTs could enhance the anti-cancer effect of gold nanoparticles with minimal side effects to normal cells [42,43]. Thus, in this study, we designed a novel platform based on gold nanostars decorated by MWCNTs, which was expected to enhance the NIR light absorption for PTT. Using an improved two-step reduction method, the hybrid MWCNTs/gold nanostars material was synthesized without requiring any surfactant. We then investigated the light-to-heat conversion efficiency of the hybrid nanomaterial and its efficiency for cancer cells ablation *in vitro* to explore its potential application for PTT.

## 2. Materials and reagents

MWCNTs with a diameter of 20–40 nm and length of 5–15 µm were obtained from Nanotech Port Co., Ltd (Shenzhen, China). Chloroauric acid (HAuCl_4_ · 4H_2_O, Au ≥ 47.8%) and PEG_5000_-SH (95%) were purchased from Sigma-Aldrich (Beijing, China). Silver nitrate (AgNO_3_) was obtained from Shanghai Chemical Reagent Co., Ltd (Shanghai, China). CTAB, tri-sodium citrate (Na_3_C_6_H_5_O_6_), hydrochloric acid (HCl), sulfuric acid (H_2_SO_4_, 98%), nitric acid (HNO_3_, 65–68%), sodium hydroxide (NaOH) and ascorbic acid were purchased from Sinopharm Group Co. Ltd. All of the above-mentioned analytical-grade materials were used without further treatment.

### 2.1. Preparation of gold nanoparticles

The MWCNTs were dealt with a simple method [44]. Briefly, 99.8 mg of MWCNTs was dissolved into 10 ml of solution with a volumetric ratio of H_2_SO_4_/HNO_3_ of 1: 3 under ultrasonication for 10 h at room temperature. Then, the solution was diluted with deionized water and 10 mmol l^−1^ NaOH aqueous solution until it became neutral. Then the solution was filtered with a 0.22 µm membrane and vacuum-dried at 60°C.

We prepared the MWCNTs/gold nanostars hybrid material using two different methods to compare the photothermal efficiency: a traditional method of seed-mediated growth process [39,45] and a two-step reduction method without CTAB [18]. In brief, an aqueous solution of 1% (w/v) tri-sodium citrate (8 ml) was added to 50 ml of boiling HAuCl_4_ · 4H_2_O (1 mM) under stirring. After the addition of 5 mg of MWCNTs, the mixture suspension was kept under continuous stirring for 15 min at room temperature (27°C) to obtain the MWCNTs/spherical gold seed particles. Subsequently, MWCNTs/gold nanostars were synthesized using the second reduction step. In brief, 100 µl of the MWCNTs/spherical gold seed particles was added to 10 ml of HAuCl_4_ · 4H_2_O (0.3 mM) solution under gentle stirring, followed by the addition of 100 µl of HCl (1 M), 100 µl of AgNO_3_ (4 mM) and 50 µl of ascorbic acid (0.1 M). The solution again became colourless and then became light blue after stirring for 30 min. Immediately following the colour change, the solution was rinsed by centrifugation (10 000 r.p.m.) and vacuum-dried. Then the powder of MWCNTs/gold nanostars was added into 1.5 ml of SHPEG5000 (5 µmol l^−1^) aqueous solution and stirred for 15 min. After 24 h at room temperature, the mixture was centrifuged at 10 000 r.p.m. for 15 min to obtain the MWCNTs/gold nanostars/PEG. The gold nanostars and gold nanostars/PEG were also prepared by the same method without MWCNTs. The synthesis of 60 nm gold nanospheres is presented by Yuan *et al*. [18] and that of the gold bipyramids with 110 nm macroaxis and 35 nm brachyaxis is presented by Navarro *et al*. [45].

### 2.2. Characterizations

The concentration of gold atoms in MWCNTs/gold nanostars aqueous dispersion was analysed by inductively coupled plasma–mass spectroscopy (ICP-MS, NexION 300X) and all the concentrations presented in this paper refer to the concentration of gold atoms. Transmission electron microscopy (TEM) images were obtained using a Tecnai G2 20 S-TWIN (FEI, America) transmission electron microscope operating at an acceleration voltage of 200 kV. For TEM, all samples were deposited on 300-mesh porous carbon-coated copper grids and dried overnight before examination. Ultraviolet/visible (UV-vis) spectra were obtained with an Ultrospec 3300 Pro UV-vis spectrophotometer (GE Healthcare) over the wavelength range of 450–1000 nm. The photothermal conversion efficiencies of the nanoparticles were determined by measuring the temperature increase of the aqueous solutions upon laser irradiation. In brief, the solutions of gold nanostars and MWCNTs/gold nanostars (1 mg ml^−1^) were added to different wells of a 12-well plate, and radiation was delivered using a diode laser centred at 808 nm from the top at a power density of 1 W cm^−2^. The temperature variation was tested by a probe thermometer. The nanoparticles used in the photothermal conversion experiments were prepared by the second method described above.

### 2.3. Cell culture

The mouse melanoma cell line B16F10 was obtained from the American Type Culture Collection (ATCC; Manassas, VA, USA). The cell growth medium was composed of Dulbecco modified Eagle medium (Invitrogen) supplemented with 10% (v/v) fetal bovine serum (FBS; Gibco). The cells were maintained in 1% penicillin/streptomycin (Hyclone) and cultured in a 5% CO_2_ incubator at 37°C. The culture medium was replaced every 3 days in all experiments.

### 2.4. *In vitro* cytotoxicity and photothermal efficiency

To detect the cytotoxicity of the materials, B16F10 cells were co-cultured with different concentrations of gold nanostars or MWCNTs/gold nanostars at a density of 2 × 10^4^ cells ml^−1^ in a 96-well plate (100 µl of cell suspension per well). To allow for internalization of the nanoparticles, they were incubated with the cells for 24 h, and then the free nanoparticles were washed with phosphate-buffered saline (PBS) and the cells were maintained in 10% Cell Counting Kit-8 (CCK-8) reagent with new fresh medium. After 2 h of culture, the viability of the cells was determined by measuring the optical density on a microplate system (Varioskan Lux) and compared to that of the control cells incubated with the same volume of PBS only.

To evaluate the photothermal efficiency of the nanoparticles, the cells were co-cultured with 0.32 nM of gold nanostars or MWCNTs/gold nanostars until reaching the exponential growth phase under the same condition. After 24 h of culture, the free nanoparticles were removed and the cells were maintained in fresh medium after washing with PBS. The cells were then irradiated by an NIR 808-nm laser at a power density of 1 W cm^−2^ for 3 min and cultured for another 24 h. The cell viability was then investigated by the CCK-8 assay as described above. For acridine orange/ethidium bromide (AO/EB) staining, B16F10 cells (2 × 10^5^ cells) grown in FBS-containing medium were cultured in 35 mm culture dishes and irradiated by the 808 nm laser after incubation with gold nanostars or MWCNTs/gold nanostars, washed with PBS and stained with 0.4% AO/EB blue solution (Sigma Inc.) for 3 min. Microscopic images of the cells were taken using a Zeiss 710 microscope. The nanoparticles used in the *in vitro* evaluations were also prepared by the second method.

### 2.5. Statistical analysis

Data are expressed as means and standard errors. Statistical significance was determined using analysis of variance or the Student *t*-test by the SPSS software. *p* < 0.05 was considered statistically significant.

## 3. Results and discussion

### 3.1. Characterization of gold nanostars and MWCNTs/gold nanostars

**Figure 1.**
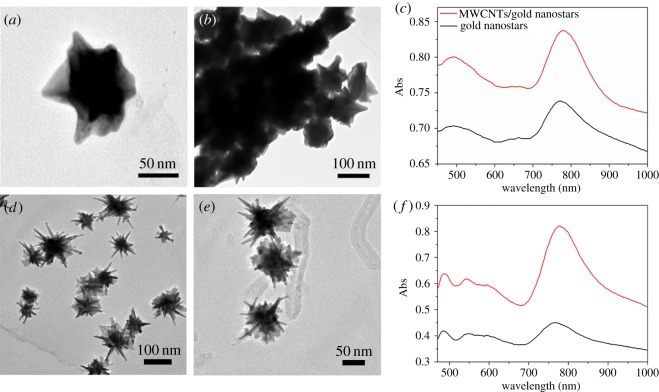
TEM images of (*a*,*d*) gold nanostars and (*b*,*e*) multiwalled carbon nanotubes (MWCNTs)/gold nanostars. (*c*,*f*) UV-visible spectrum of gold nanostars and MWCNTs/gold nanostars prepared with two different synthetic methods: the traditional method (*a*–*c*) and a two-step reduction method (*d*–*f*).

The morphology and microstructure of gold nanostars and MWCNTs/gold nanostars were investigated by TEM. Figure 1*a* depicts the TEM image of the gold nanostars prepared with the traditional method, with some branches evident; the average size of the spherical gold nanostars was approximately 70 ± 3 nm and that of the ‘tips’ was approximately 10 ± 4 nm. In the TEM image of the MWCNTs/gold nanostars, the MWCNTs are not clearly evident owing to aggregation of the gold nanostars (figure 1*b*). In the UV-vis spectrum, the resonance at 478 nm could be attributed to the spherical seeds and that at 778 nm is attributed to the ‘tips’ of the MWCNTs/gold nanostars (figure 1*c*). In addition, the MWCNTs/gold nanostars exhibited higher intensity absorption than the gold nanostars with a 5 nm red shift.

Figure 1*d* shows the TEM image of the gold nanostars prepared using the improved nanoparticle synthesis method. Similar to the nanostars prepared with the traditional method, significant branches were clearly evident; however, in this case, the branches had longer and sharper tips and the average size of the spherical gold nanostars was slightly smaller at approximately 60 ± 3 nm, whereas that of the ‘tips’ was larger at approximately 35 ± 4 nm. Figure 1*e* shows the TEM image of the MWCNTs/gold nanostars prepared with the second method, demonstrating that the gold nanostars were clearly loaded on the surface of the MWCNTs. In the UV-vis spectrum, the resonance at 480 nm is attributed to the spherical seeds and that at 820 nm is attributed to the ‘tips’ of MWCNTs/gold nanostars (figure 1*e*). Again, the MWCNTs/gold nanostars showed higher absorption intensity than the gold nanostars with a 7 nm red shift.

For gold nanoparticles to be ideally used in biological applications, they should be of low toxicity with good biocompatibility, induce minimal side effects and plasmon-tunability in the NIR region [24]. Traditional synthesis methods require the use of CTAB, which is not only potentially toxic to humans but is also difficult to replace during functionalization and to wash off. By contrast, the synthesis approach developed in this study shows the following advantages compared to the traditional method: (i) the preparation method is more convenient with low toxicity; (ii) the gold nanostars have an improved morphology with longer branches of similar size; (iii) the aggregation phenomenon of gold nanostars is obviously reduced (figure 1*b*,*e*); and (iv) there is a small 42 nm red shift absorption of the UV-vis spectrum. Collectively, these results demonstrate that the proposed method is more useful and advantageous for preparing gold nanoparticles for biomedical applications.

### 3.2. Optical properties of gold nanostars and MWCNTs/gold nanostars

**Figure 2.**
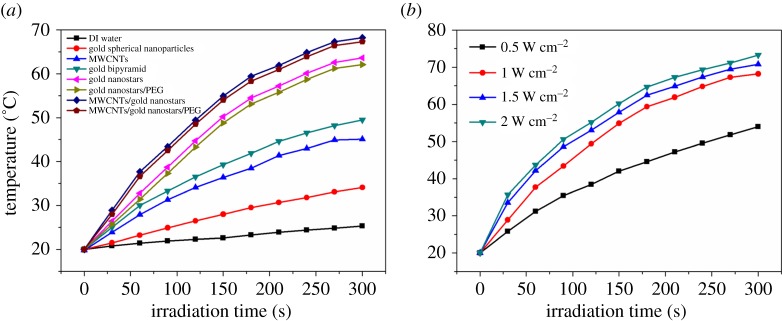
The heating curves of (*a*) deionized (DI) water, gold spherical nanoparticles, MWCNTs, gold bipyramid, gold nanostars, gold nanostars/PEG, MWCNTs/gold nanostars and MWCNTs/gold nanostars/PEG under 808 nm laser irradiation at a power density of 1.0 W cm^−2^ and (*b*) MWCNTs/gold nanostars under 808 nm laser irradiation at different power density; the concentration of the materials was 4.9 nM.

The photothermal conversion efficiency of the nanoparticles is a significant factor to consider for their biological applications. Therefore, we determined the temperature profiles of the gold nanostars and MWCNTS/gold nanostars by directly irradiating aqueous solutions of the nanoparticles with lasers and measuring the temperature changes with a probe thermometer. As shown in figure 2*a*, the temperature of the gold nanostars and gold nanostars/PEG solutions underwent a rapid increase from 20°C to more than 62°C in 5 min under stimulation of an 808 nm laser at a power density of 1.0 W cm^−2^, and the heating rate reached 8.72°C min^−1^ and 8.42°C min^−1^, respectively. By contrast, the temperature changes of the gold nanospheres and gold bipyramids solutions were much less dramatic under the same laser irradiation conditions. More importantly, the heating rates of the MWCNTs/gold nanostars and MWCNTs/gold nanostars/PEG solutions were both higher than those of the other nanoparticles, reaching up to 9.46°C min^−1^ and 9.64°C min^−1^, respectively. This result demonstrated that the hybrid nanomaterial has greatly improved photothermal conversion efficiency in comparison with that of gold-based nanomaterials previously developed by Qiu *et al.* [7] and Lee *et al.* [46], which is probably attributed to the increased light absorption conferred by the MWCNTs in combination with the plasmonic effects from the gold nanostars. We then tested the profiles of MWCNTs/gold nanostars with different power densities. As shown in figure 2*b*, there is no significant temperature increase when we changed the power density to 1.5 W cm^−2^ and 2 W cm^−2^. However, when the power density decreased to 0.5 W cm^−2^, the temperature increase had a dramatic decrease. Considering the biosafety, we chose 1.0 W cm^−2^ in our *in vitro* experiment.

### 3.3. Cytotoxicity *in vitro*

**Figure 3.**
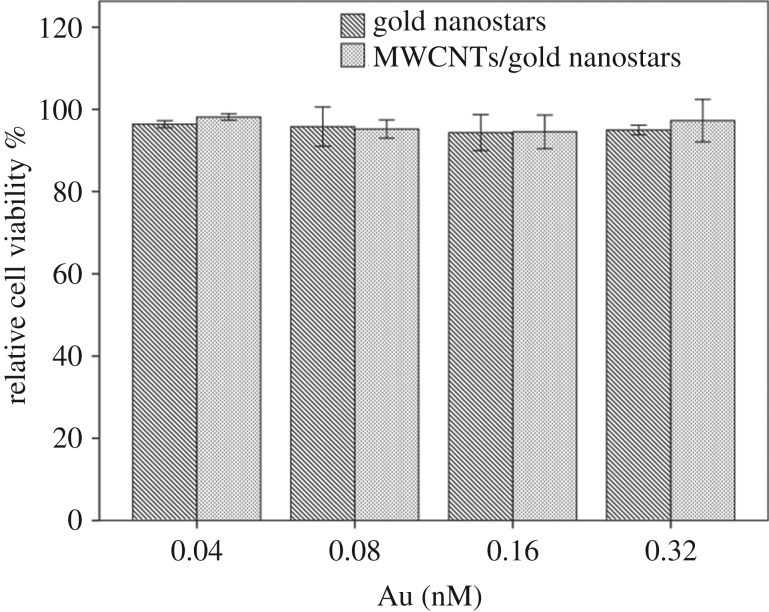
Relative viability of B16-F10 cells incubated with different concentrations of gold nanostars and MWCNTs/gold nanostars. Data are presented as means ± standard errors (*n* = 6).

Before subjecting the nanomaterials to biological experiments, it is important to first determine their baseline cytotoxicity using standard cell toxicity tests. The toxicity of the gold nanostars and MWCNTs/gold nanostars was assessed by the CCK-8 assay with the melanoma cell line B16F10. The viabilities of B16F10 cells were determined after 24 h incubation with the gold nanostars and MWCNTs/gold nanostars at different concentrations ranging from 0.04 nM to 0.32 nM [43]. As shown in figure 3, no significant cytotoxicity was observed for either the gold nanostars or MWCNTs/gold nanostars even at the highest concentration. This could be attributed to the intrinsic non-toxic nature of gold and MWCNTs, as well as the lack of toxic chemical reagents and surfactants used in the synthesis process.

### 3.4. *In vitro* photothermal therapy of MWCNTs/gold nanostars

**Figure 4.**
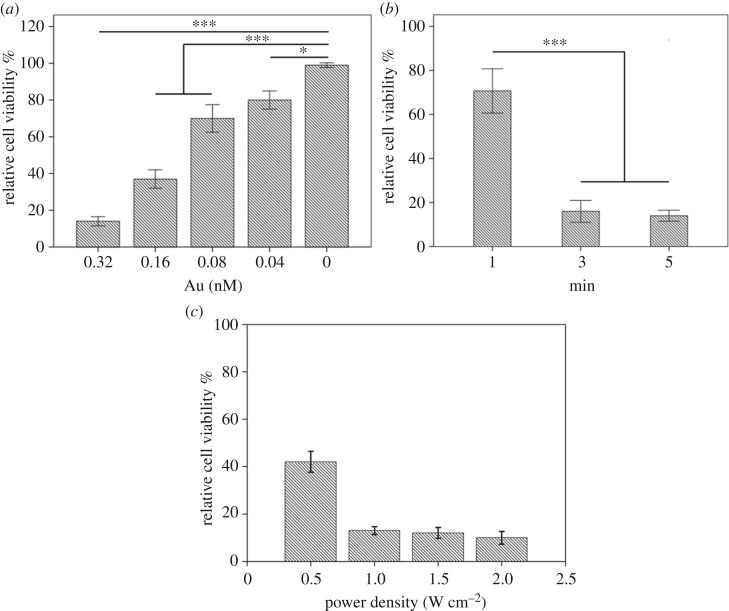
Relative viability of B16-F10 cells incubated with (*a*) different concentrations of gold MWCNTs/gold nanostars after irradiation by an 808 nm laser (1.0 W cm^−2^, 3 min), (*b*) 0.32 nM MWCNTs/gold nanostars for different irradiation times (1.0 W cm^−2^) and (*c*) 0.32 nM MWCNTs/gold nanostars with different power density (3 min). Each value represents the mean ± standard error (*n* = 6). *p < 0.05, **p < 0.01, ***p < 0.001.

**Figure 5.**
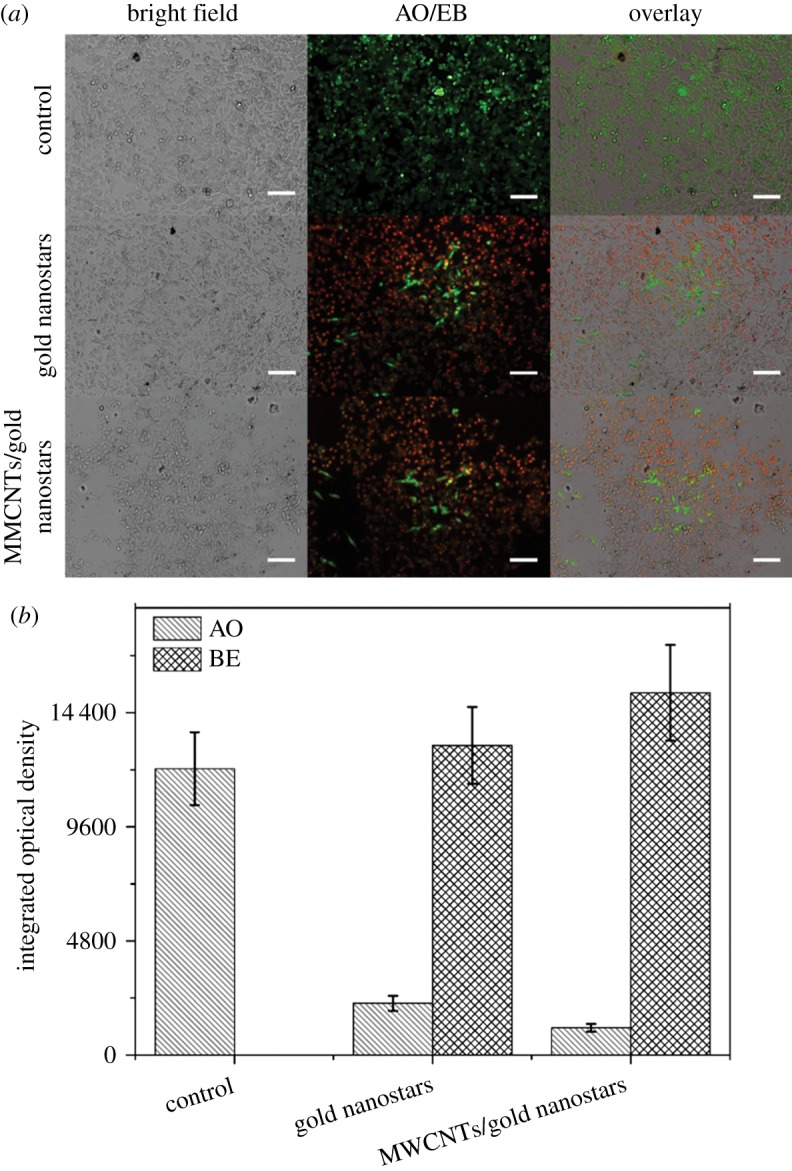
(*a*) Fluorescence images of AO/EB-stained B16F10 cells incubated with a control condition, gold nanostars and MWCNTs/gold nanostars at the same concentration of 0.32 nM. Scale bars, 100 µm. (*b*) Fluorescence intensity of AO/EB. Data are presented as means ± standard errors (*n* = 5).

Finally, to explore the ability of the hybrid material for enhanced photothermal ablation of cancer cells, different concentrations of MWCNTs/gold nanostars were co-cultured with B16F10 melanoma cells for 24 h when the cells reached the exponential growth phase, and then the cells were irradiated with an 808 nm laser for 3 min. Photothermal laser treatment was initiated with a power density of 1.0 W cm^−2^ (figure 4*a*) [43]. As the greatest ablation efficiency of the materials was achieved at the highest concentration, we chose a concentration of 0.32 nM for this experiment, which was the optimum concentration without any toxic side effects to the cells. The optimal irradiation time and power density of the laser were also determined to be 3 min and 1.0 W cm^−2^, respectively, which almost achieve the greatest ablation of cells, as shown in figure 4*b* and Figure 4*c*. To evaluate the ability for targeted cancer cell ablation, confocal fluorescence microscopy images of AO/EB co-stained cells were observed after treatment under the optimal condition (figure 5*a*), and the fluorescence intensity of AO/EB was calculated (figure 5*b*). The MWCNTs/gold nanostars showed greater photothermal efficiency than the gold nanostars, which resulted in many cancer cells detaching from the substrate.

**Figure 6.**
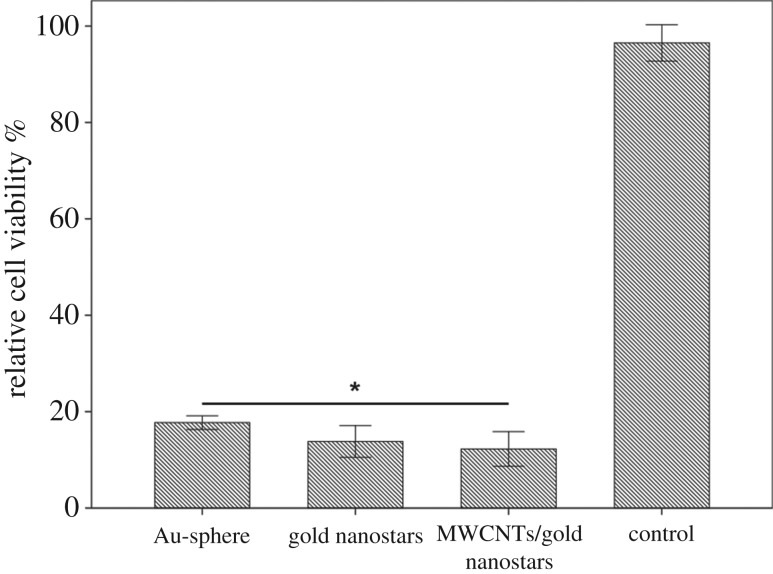
Relative viability of B16-F10 cells incubated with 0.32 nM gold nanospheres, gold nanostars, MWCNTs/gold nanostars and nanoparticle-free media after irradiation with an 808 nm laser (1.0 W cm^−2^, 3 min). Data are presented as means ± standard errors (*n* = 6). *p < 0.05.

To further demonstrate the superior photothermal efficiency of the MWCNTs/gold nanostars composite over other types of gold nanostructures, we also compared its ability of cancer cell ablation with that of conventional gold nanospheres. As shown in figure 6, under the same power density, all three materials produced more than 80% cell death. However, both the gold nanostars and MWCNTs/gold nanostars showed greater photothermal efficiency than the gold nanospheres, which are widely used in the design of photothermal materials. This result demonstrated that the presence of ‘tips’ on the surface of gold nanostars can promote the photothermal efficiency of the nanomaterial. Moreover, cells treated with the MWCNTs/gold nanostars showed the greatest amount of cell death, demonstrating the added benefit of MWCNT decoration.

## 4. Conclusion

Anisotropic gold nanoparticles exhibit significant enhancement in surface plasmon resonance in the NIR range, particularly when designed in a star shape. Moreover, for biological applications, a convenient and green synthesis method is required. To meet these needs, in the present study, we synthesized a biocompatible MWCNTs/gold nanostars hybrid nanomaterial using an improved two-step reduction method by controlling several synthetic conditions such as the pH value, concentrations of reducing agent and some other reagents. We also prepared gold nanostars alone using a similar reducing agent without any harsh surfactant. This two-step reduction method generated gold nanostars with longer branches of a similar size, and improved the dispersion of nanostars in the MWCNTs/gold nanostars hybrid. The UV-vis spectra also indicated an obvious red shift in the NIR range. Importantly, the MWCNTs/gold nanostars exhibited dramatically improved photothermal conversion efficiency and cancer cell ablation over gold nanostars alone and the more conventionally used gold nanospheres, which could be attributed to the strong surface plasmon resonance absorption of the gold nanostars decorated on the MWCNTs with significantly elongated tips. Thus, the combination of favourable properties of this composite material highlights the great potential of the MWCNTs/gold nanostars developed in our study as a promising nanoplatform for PTT to improve cancer therapy.

## Data accessibility

This article has no additional data.
